# Dynamic CT-based assessment of ulnar-sided wrist kinematics in healthy participants using automated 3-D analysis

**DOI:** 10.1177/17531934251397297

**Published:** 2025-11-21

**Authors:** Erin Teule, Jesse Wolters, Nico Verdonschot, Stefan Hummelink, Brigitte van der Heijden

**Affiliations:** 1 Department of Plastic Surgery, Radboud University Medical Center, Nijmegen, The Netherlands; 2 Orthopaedic Research Laboratory, Radboud University Medical Center, Nijmegen, The Netherlands; 3 Technical Medicine, University of Twente, Enschede, The Netherlands; 4 Laboratory of Biomechanical Engineering, University of Twente, Enschede, The Netherlands; 5 Department of Plastic Surgery, Jeroen Bosch Hospital, Hertogenbosch, The Netherlands

**Keywords:** automated 3-D analysis, distal radioulnar joint, DRUJ, dynamic CT, TFCC, ulnar-sided wrist pain, wrist, wrist kinematics

## Abstract

**Introduction:**

Dynamic CT imaging may help to diagnose ulnar-sided wrist pain by analysing joint motion in normal and pathological wrists. In this study an automated 3-D workflow was developed to quantify ulnocarpal impaction and distal radioulnar joint stability with dynamic CT imaging in normal wrists during motion. The aim was to establish normal values which can be used as a reference.

**Methods:**

Dynamic CT scans were acquired from the dominant wrist of 59 healthy participants during three wrist movements: flexion–extension and radioulnar deviation were measured in 30 wrists and pronation–supination in 29. Ulnocarpal impaction was assessed with 3-D ulnar variance, reflecting proximal–distal translation of the distal radioulnar joint, and ulnocarpal proximity to the lunate and triquetrum. Distal radioulnar joint stability was evaluated using the 3-D epicentre and 3-D modified radioulnar line methods, which captured palmar–dorsal translation.

**Results:**

Minimal proximal–distal and palmar–dorsal translation of the distal radioulnar joint was observed across all wrist movements, although considerable inter-individual variation was notable. Three-dimensional ulnar variance did not increase during pronation. Ulnocarpal proximity to the triquetrum showed greater variation during wrist motion than to the lunate (median range 4.2–5.0 vs. 1.2–1.4 mm, respectively). Palmar–dorsal translation was larger during pronation–supination (3-D epicentre range 15.2%) than during flexion–extension and radioulnar deviation (7.7 and 9.3%, respectively).

**Conclusion:**

Dynamic CT imaging shows promise for evaluating the distal radioulnar joint during wrist motion. Normal values that quantify ulnocarpal impaction and distal radioulnar joint stability found in this study may serve as a reference for future research of ulnar-sided wrist pathology.

**Level of evidence:**

IV

## Introduction

Ulnar-sided wrist pain can be difficult to diagnose owing to the complex anatomy and biomechanics of the wrist. Common dynamic ulnar wrist disorders include distal radioulnar joint (DRUJ) instability and ulnocarpal impaction syndrome (Brogan et al., 2019; DaSilva et al., 2017). Distal radioulnar joint instability results from soft tissue insufficiency, particularly of the dorsal and palmar ligaments of the triangular fibrocartilage complex, and may lead to painful subluxation of the ulna (Mirghasemi et al., 2015; Saito et al., 2017). Ulnocarpal impaction occurs when the ulna abuts the carpus during wrist motion, potentially causing degenerative changes to the lunate and triquetrum (Brogan et al., 2019). Both DRUJ instability and ulnocarpal impaction may be missed with conventional static imaging techniques (Brogan et al., 2019; Squires et al., 2014). Assessment of ulnocarpal impaction could be improved by measuring ulnar variance (UV) using 2-D dynamic fluoroscopy; however, its diagnostic value is hampered by projection errors, beam angulation dependency, and the inability to reliably quantify wrist motion (Epner et al., 1982; Squires et al., 2014). In this context, dynamic, 3-D imaging can be helpful for more accurately diagnosing ulnar-sided wrist pain.

A detailed understanding of normal ulnar-sided wrist kinematics can help the diagnosis and treatment of related pathologies. Previous studies have analysed DRUJ stability using static, 2-D CT-based measurements, showing physiological translation of the radius relative to the ulna during pronation and supination (Park et al., 2024; Wijffels et al., 2016). However, these studies relied on static imaging and/or analysed small cohorts, limiting its clinical relevance. Moreover, studies using dynamic CT imaging relied to a great extent on manual analysis, which may be subjective and time-consuming (Carr et al., 2019; Oonk et al., 2023; Shakoor et al., 2019).

With recent advances in imaging and automated analysis, large-scale and objective assessment is now feasible. In this study, we developed a fully automatic software-based workflow to quantify DRUJ instability and ulnocarpal impaction in normal wrists based on dynamic CT imaging. The aim of this study was to automatically quantify 3-D CT radiological parameters of normal wrists during motion that can serve as a reference for future studies of dynamic ulnar-sided wrist disorders, including DRUJ instability and ulnocarpal impaction syndrome.

## Methods

### Participants

This study is part of a large-scale dynamic CT research project investigating wrist joint kinematics and is conducted at the Radboud University Medical Centre (Nijmegen, the Netherlands). The dynamic CT data analysed in the present study consist of two parts. The first part comprised dynamic CT scans of 30 healthy participants performing radioulnar deviation (RUD) and flexion–extension (FE). Ethical approval was obtained for the collection of this dataset from the institutional review board (ABR number NL72518.091.19) and it was used in other publications addressing different research objectives (Haenen et al., 2025a; Haenen et al., 2025b; Teule et al., 2024a). For the present study, an additional dataset was collected from a separate group of 30 healthy participants performing pronation–supination (PS), for which separate approval from the institutional review board was obtained (ABR number NL84487.091.23). The inclusion and exclusion criteria for both datasets were the same. Eligible participants were healthy participants aged 18–65 years old, without a medical history of wrist trauma, pain or surgery and with full wrist mobility. Exclusion criteria included pregnancy and the presence of osteoarthritis on the static CT scan. All participants provided written informed consent.

### Dynamic CT scan procedure and analysis

Each dynamic CT dataset included a static scan followed by dynamic scans acquired using an Aquilion ONE Prism CT system (Canon Medical Systems, Otawara, Japan). The static scan captured the ulna, radius and all carpal bones with the participant in prone position and the forearm and wrist in neutral position. Static scans were acquired with 80 kV and automatic mA calculation and processed using advanced image reconstruction software (Advanced intelligent Clear-IQ Engine, Canon Medical Systems) optimized for bone detail. This resulted in high-resolution images with a voxel size of 0.50 mm in all dimensions.

The dynamic scan protocol differed between the two aforementioned datasets from which imaging data were used. In the first protocol unilateral dynamic CT scans of the dominant wrist were acquired in 30 healthy participants during RUD and FE, each lasting 7 s. In the second protocol bilateral dynamic CT scans in a separate group of 30 healthy participants were acquired during PS in 10 s. Based on the low radiation exposure with the initial imaging protocol, the medical ethics committee approved bilateral scanning in the second data collection. For the present study, only CT data of dominant wrists from both datasets were used to maintain consistency, as the initial dataset included scans of dominant wrists only.

**Figure 1.**
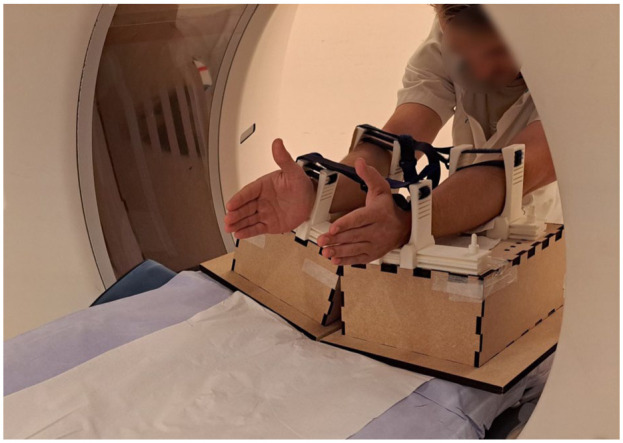
Set-up for dynamic CT scanning. The patient is positioned at the back end of the gantry. Both forearms are supported by a frame to minimize forearm motion during image acquisition.

To minimize motion artefacts and standardize wrist movement, participants practised movements before imaging, and both forearms were supported in a frame during acquisition (Figure 1). The field of view in axial direction was limited to 8 cm to include all carpal bones and the DRUJ while minimizing radiation exposure. CT settings were consistent in both studies (80 kV, 40 mA). All dynamic scans were reconstructed at 10 scans per second using the same image reconstruction software and a body sharp kernel, with an average voxel size of 0.59 × 0.59 × 0.50 mm. The CT dose index of the entire imaging protocol was approximately 7 mGy in the first protocol and 5 mGy in the second, with dose–length products of about 65 and 55 mGy·cm, respectively. The total effective dose remained below 0.1 mSv in both imaging protocols.

Analysis of the dynamic CT scans was performed using previously developed, fully automatic analysis software (Haenen et al., 2025b). First, the ulna, radius and all carpal bones were automatically segmented using a 3-D no-new U-net architecture model, achieving an accuracy (Dice score) of 95.9% (SD 2.1%), indicating excellent performance (Teule et al., 2024b). Segmented bones from the static scan were subsequently registered to the corresponding dynamic positions. Local coordinate systems were automatically determined in the radius, ulna and carpal bones to enable extraction of wrist kinematics (Haenen et al., 2025a).

### Parameter description

**Figure 2.**
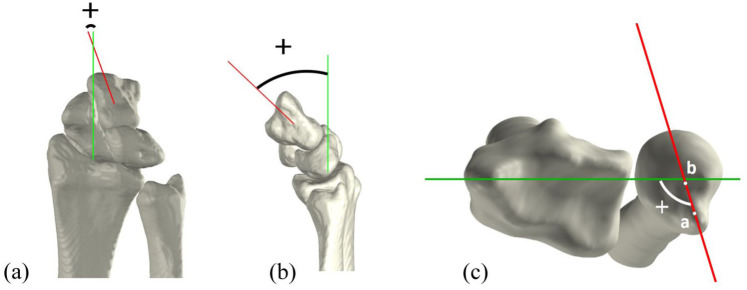
Definition of wrist angles. (A) Coronal view of a radially deviated wrist, depicting the coronal capitoradial (CR) angle between the axis of the radius (green) and axis of the capitate (red). (B) Sagittal view of a flexed wrist, depicting the sagittal CR angle between the axis of the radius (green) and the axis of the capitate (red). (C) Axial view of a pronated wrist, depicting the ulnoradial angle between the mediolateral axis of the radius (green) and axis of the ulna (red), connecting the ulnar styloid (a) and the ulnar epicentre (b).

Four 3-D parameters were developed to measure ulnocarpal impaction and DRUJ instability using Matlab (v.R2022b, MathWorks, Natick, MA, USA). Ulnocarpal impaction was quantified using the 3-D ulnar variance (3-D UV) (Steyers and Blair, 1989) and ulnocarpal proximity (UcP), while DRUJ instability was assessed using the 3-D epicentre method (3-D epi) and the 3-D modified radioulnar line method (3-D mru) (Park and Kim, 2008; Shakoor et al., 2019). Detailed explanations of these parameters are provided in the following paragraphs. All parameters were plotted relative to the wrist angle, defined as the sagittal capitoradial (CR) angle during FE, the coronal CR angle during RUD, and the ulnoradial angle in the axial plane during PS (Figure 2). For the 3-D UV, 3-D epi and 3-D mru, the delta value, a measure of the difference between two values, was also calculated. The delta value was defined as the change relative to the neutral wrist position. The normality of all parameters across wrist angles was evaluated using the Shapiro–Wilk test. Since several parameters did not meet the criteria for normal distribution, data are reported as median values with corresponding interquartile ranges (IQRs).

### Ulnocarpal impaction: 3-D ulnar variance

**Figure 3.**
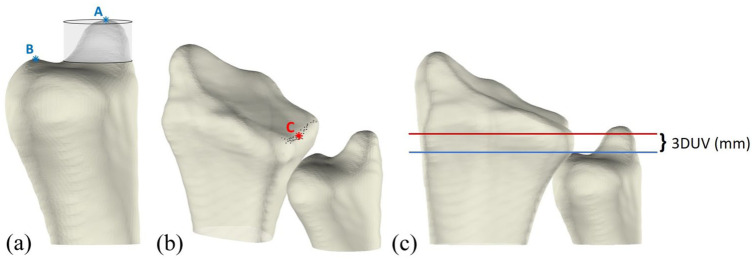
Illustration of 3-D ulnar variance (UV) measurement. (A) Identification of the distal ulnar articular surface. The transparent cylinder surrounding the ulnar styloid process (A) is removed from the bone, after which the most distal point (B) is determined. (B) Identification of most distal point (C) in the middle of the sigmoid notch. (C) Coronal view of the radius and ulna, with two lines in the coronal plane: a red line through point C and a blue line through point B. The 3-D UV is calculated as the height difference between these two lines.

Three-dimensional UV was defined as the relative height difference between the most distal point of the ulnar head articular surface and the most distal point of the radial sigmoid notch, as previously described (Steyers and Blair, 1989). To enable automated measurements, several anatomical landmarks were first identified automatically. The ulnar styloid was removed from the ulnar bone, and the most distal point of the remaining articular surface was identified (Figure 3A). To determine the radial point, the mean curvature of the radial notch in the longitudinal direction was calculated. Owing to the greater curvature of the sigmoid notch relative to the surrounding bone of the distal radius, points with curvature values exceeding 0.1 were classified as part of the sigmoid notch, with the most distal point designated as the radial point (Figure 3B). The 3-D UV was then determined by defining two reference lines in the coronal plane (one passing through the ulnar point and the other through the radial point) and measuring the height difference between these lines along the longitudinal axis (Figure 3C).

### Ulnocarpal impaction: ulnocarpal proximity

**Figure 4.**
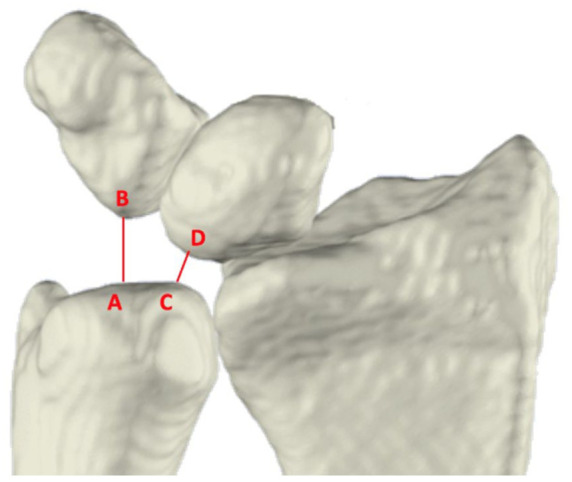
Anterior view of the radius, ulna, lunate, and triquetrum. Ulnocarpal proximity is the shortest distance between the ulna and the triquetrum (A, B) and between the ulna and the lunate (C, D).

The UcP was defined as the shortest 3-D distance between the ulna and either the lunate (UcP-L) or triquetrum (UcP-T). These measurements were automatically obtained from the segmented wrist bones (Figure 4). Because the shortest distance can occur at different anatomical sites, the exact measurement location may vary between wrist positions.

### DRUJ stability: 3-D modified radioulnar line method

**Figure 5.**
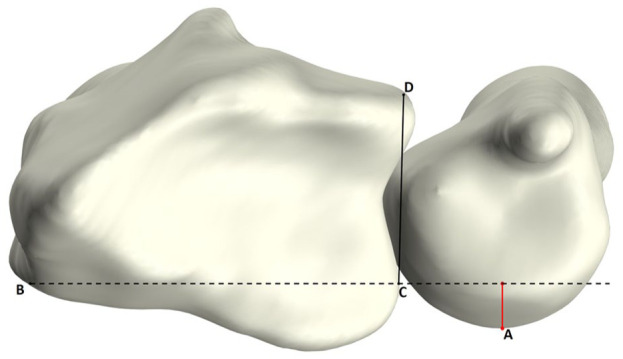
Axial view of the radius and ulna illustrating the 3-D modified radioulnar (mru) line measurement. Point A: most palmar point of the ulnar head; point B: palmar–radial corner; point C: palmar margin of sigmoid notch; point D: dorsal margin of sigmoid notch. Line B–C (black dashed line) represents the palmar–radial line. The 3-D mru is the ratio of the perpendicular distance between A and line B–C (red line) to the width of the sigmoid notch (C–D; black solid line).

The 3-D mru was used to estimate ulnar stability by calculating the protrusion of the ulnar head beyond the sigmoid notch relative to its width (Figure 5), as originally described by Mino et al. (1985). Anatomical landmarks on the distal radius were automatically defined using a previously established method involving a statistical shape model of the radius (Haenen et al., 2025a). These landmarks included the palmar–radial corner and dorsal and palmar margins of the sigmoid notch (points B, C, and D in Figure 5). Next the palmar–radial line was drawn connecting the palmar–radial corner and palmar margin of the sigmoid notch. Ulnar head protrusion was calculated as the shortest distance between the most palmar point on the ulnar head (Figure 5, point A) and the palmar–radial line (Figure 5, black dashed line). The 3-D mru was calculated as the percentage of this protrusion distance (Figure 5, red line) to the sigmoid notch width (Figure 5, black solid line). Dorsal dislocation of the radius relative to the ulnar head was considered positive, while palmar dislocation of the radius was considered negative.

### DRUJ stability: 3-D epicentre method

**Figure 6.**
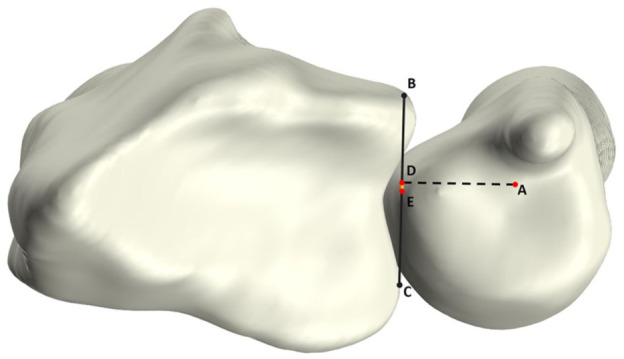
Axial view of the radius and ulna. Illustration of the 3-D epicentre (epi) measurement, which is defined by projecting a point (D) from the origin of the longitudinal ulnar axis (A) on the sigmoid notch (B–C; black line). The 3-D epi is calculated as the ratio of the distance from D to the midpoint of the sigmoid notch (D–E; yellow line), and the sigmoid notch width (B–C).

Three-dimensional epi, also used to assess ulnar stability, was derived by calculating the ratio of the palmar-to-dorsal distance from the sigmoid notch midpoint and the ulnar epicentre to the width of the sigmoid notch (Figure 6). As in the 3-D mru method, a statistical shape model was used to automatically determine the sigmoid notch width and its midpoint (Figure 6, points B, C and E). First the longitudinal axis of the ulna was automatically computed. The intersection of this axis with the distal cortex was defined as the ulnar epicentre (Figure 6, point A). Subsequently, the projection of the ulnar epicentre onto the sigmoid notch line was calculated (Figure 6, point D). The 3-D epi was then calculated as the percentage of the projected ulnar epicentre to sigmoid notch midpoint distance (Figure 6, D-E) relative to the sigmoid notch width (Figure 6, B-C). Similar to the 3-D mru, relative dorsal dislocation of the radius was considered positive, while palmar dislocation was considered negative.

## Results

Initially, a total of 60 wrists were included in this study, of which 30 performed RUD and FE movements and the other 30 performed a PS movement during dynamic CT imaging. Owing to a scanning error, one PS movement had to be excluded, resulting in a final dataset of 59 wrists comprising 30 RUD, 30 FE, and 29 PS movements. Of these 59 wrists, 34 were women (58%). Participants had a mean age of 31 years (SD 10). As only the dominant wrist was analysed, 56 wrists (95%) were right-sided.

### Ulnocarpal impaction: 3-D ulnar variance

**Figure 7.**
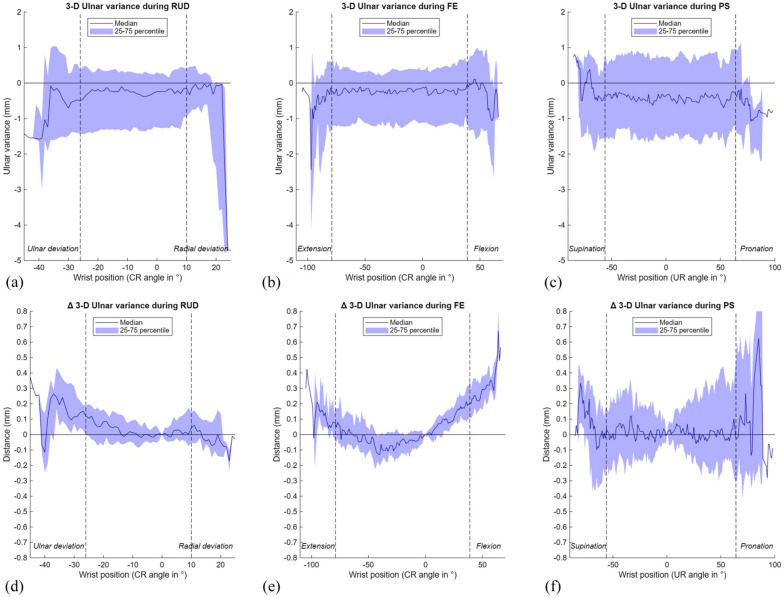
Results of the 3-D ulnar variance (UV) during three wrist movements. The range of motion in between the vertical dotted lines is achieved by a minimum of 22 wrists. (A) 3-D UV during radioulnar deviation (RUD); (B) 3-D UV during flexion–extension (FE); (C) 3-D UV during pronation-supination (PS); (D) Δ3-D UV during RUD; (E) Δ3-D UV during FE; (F) Δ3-D UV during PS. CR: capitoradial; UR: ulnoradial.

Across all three wrist movements, our cohort showed a slightly negative median 3-D UV, remaining within the neutral range (<1 mm) (Table 1, Figure 7A–C). The change in 3-D UV relative to the neutral position (Δ3-D UV) was minimal during RUD, suggesting consistent height differences between the radius and ulna. Larger variations were observed during FE and PS, with a non-linear trend during FE and greater inter-individual variability during PS (Figure 7D–F).

**Table 1.** Ulnar sided 3-D CT parameters in neutral and extreme positions of the wrists, with ranges (i.e. maximum minus minimum value) per wrist movement for all parameters. Data are reported as median (IQR).

Imaging and wrist movement type		3-D ulnar variance (mm)	3-D ulnocarpal proximity triquetrum (mm)	3-D ulnocarpal proximity lunate (mm)	3-D epicentre (%)	3-D modified radioulnar line (%)
Static imaging (*n* = 59)	Neutral wrist position	0.4 (−1.5 to 0.3)	6.9 (6.0 to 8.1)	3.2 (2.6 to 3.8)	−7.5 (−13.7 to 2.2)	−2.3 (−11.1 to 8.9)
Dynamic imaging: radioulnar deviation (*n* = 30)	25° ulnar deviation	−0.4 (−1.4 to 0.5)	3.8 (3.0 to 5.1)	3.3 (2.7 to 4.2)	0.4 (−8.5 to 6.1)	3.0 (−8.1 to 13.6)
	10° radial deviation	−0.1 (−0.9 to 0.4)	8.2 (7.3 to 9.6)	2.9 (2.3 to 3.4)	−1.9 (−9.3 to 3.5)	−2.6 (−8.5 to 10.1)
	Range	0.4 (0.3 to 0.5)	5.0 (4.1 to 6.6)	1.3 (0.7 to 3.0)	9.3 (6.2 to 11.4)	8.9 (6.6 to 13.2)
Dynamic imaging: flexion–extension (*n* = 30)	60° extension	−0.2 (−1.2 to 0.4)	5.4 (4.8 to 6.6)	2.6 (2.1 to 3.4)	−4.1 (−9.6 to 2)	−0.1 (−8.1 to 8.0)
	40° flexion	−0.1 (−1.3 to 0.6)	7.3 (6.2 to 7.9)	3.6 (2.8 to 4.7)	−4.9 (−10.9 to 5.4)	1.3 (−9.7 to 18.2)
	Range	0.6 (0.5 to 0.8)	4.5 (3.6 to 5.0)	1.2 (0.9 to 1.6)	7.7 (4.8 to 9.1)	8.1 (4.8 to 9.9)
Dynamic imaging: pronation–supination (*n* = 29)	50° supination	−0.4 (−1.6 to 0.7)	7.0 (6.4 to 8.1)	3.2 (2.4 to 3.9)	0.2 (−8.9 to 7.6)	11.9 (−2.8 to 20.3)
	50° pronation	−0.5 (−1.6 to 0.4)	5.1 (4.2 to 6.2)	3.1 (2.4 to 3.9)	−10.0 (−19.8 to −3.9)	−11.2 (−20.8 to −0.2)
	Range	1.1 (0.7 to 1.5)	4.2 (3.5 to 4.9)	1.4 (0.8 to 1.5)	15.2 (11.6 to 22.8)	28.6 (24.1 to 32.2)

### Ulnocarpal impaction: ulnocarpal proximity

**Figure 8.**
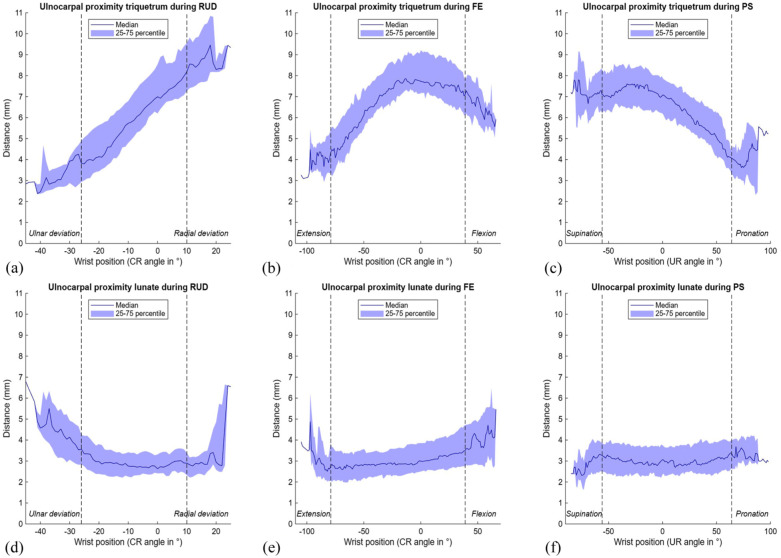
Ulnocarpal proximity to the triquetrum and lunate during three wrist movements. The range of motion in between the vertical dotted lines is achieved by a minimum of 22 wrists. (A) Ulnocarpal proximity to the triquetrum during radioulnar deviation (RUD); (B) ulnocarpal proximity to the triquetrum during flexion-extension (FE); (C) ulnocarpal proximity to the triquetrum during pronation-supination (PS); (D) ulnocarpal proximity to the lunate during RUD; (E) ulnocarpal proximity to the lunate during FE; (F) ulnocarpal proximity to the lunate during PS. CR: capitoradial; UR: ulnoradial.

Ulnar proximity to the triquetrum was generally greater than to the lunate, and varied more during wrist motion (Table 1, Figure 8). Median UcP-T decreased markedly from 8.2 mm (7.3 to 9.6) in radial deviation to 3.8 mm (3.0 to 5.1) in ulnar deviation, while during FE, the smallest values were observed in wrist extension (5.4 mm (4.8 to 6.6)). During PS, median UcP-T increased from 5.1 mm (4.2 to 6.2) in pronation to 7.0 mm (6.4 to 8.1) in supination (Table 1, Figure 8 A–C). In contrast, median UcP-L remained relatively constant during FE and PS (approximately 3 mm), while during RUD this parameter slightly decreased from 3.3 mm (2.7 to 4.2) in ulnar deviation to 2.9 mm (2.3 to 3.4) in radial deviation (Table 1, Figure 8D–F). Supplementary videos online (files 1–3) illustrate both impaction parameters and dynamic 3-D visualisations for all movements.

### DRUJ instability: 3-D modified radioulnar line method

**Figure 9.**
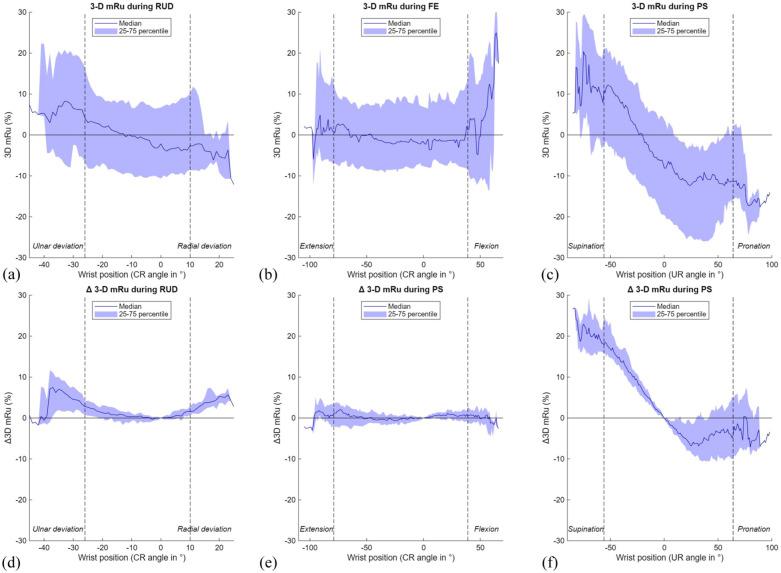
Results of the 3-D modified radioulnar (mru) line method during three wrist movements. The range of motion in between the vertical dotted lines is achieved by a minimum of 22 wrists. (A) 3-D mru during radioulnar deviation (RUD); (B) 3-D mru during flexion-extension (FE); (C) 3-D mru during pronation-supination (PS); (D) Δ 3-D mru during RUD; (E) Δ 3-D mru during FE; (F) Δ 3-D mru during PS. CR: capitoradial; UR: ulnoradial.

Using the 3-D mru method, palmar-dorsal translation of the DRUJ remained stable during FE and RUD for both the normal and Δ3-D mru, but showed a consistent shift from a median of −11.2% (−20.8 to −0.2) in pronation to 11.9% (−2.8 to 20.3) in supination (Table 1, Figure 9), indicating relative palmar protrusion of the radius in pronation and dorsal protrusion in supination. This suggests that translation of the radius relative to the ulnar head is most pronounced during PS and only minimal during RUD and FE.

### DRUJ instability: 3-D epicentre method

**Figure 10.**
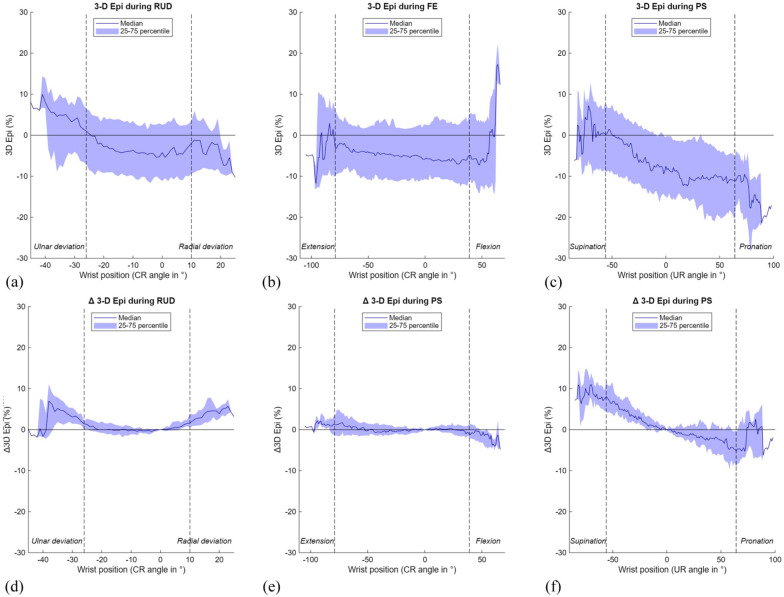
Results of the 3-D epicentre (epi) method during three wrist movements. The range of motion in between the vertical dotted lines is achieved by a minimum of 22 wrists. (A) 3-D epi during radioulnar deviation (RUD); (B) 3-D epi during flexion-extension (FE); (C) 3-D epi during pronation-supination (PS); (D) Δ 3-D epi during RUD; (E) Δ 3-D epi during FE; (F) Δ 3-D epi during PS. CR: capitoradial; UR: ulnoradial.

The 3-D epi results mirrored those of the 3-D mru method, with minimal changes during FE and RUD and a similar palmar-to-dorsal shift of the radius during PS (Table 1, Figure 10). Despite this variation, both methods indicated that the ulnar head remained well contained within the sigmoid notch throughout motion. Videos illustrating both DRUJ kinematic parameters and a 3-D visualisation during FE, RUD and PS in a normal wrist have been included in the supplementary materials online (Supplementary files 4-6).

## Discussion

In this study a novel 3-D approach is presented to analyse ulnar-sided wrist kinematics using dynamic CT and normal values during wrist motion were provided. While 3-D dynamic CT has advanced for evaluating radial-sided wrist kinematics (van der Heijden et al., 2025), its application to the ulnar-sided wrist remains limited. Our automated approach improved the understanding of ulnar-sided wrist kinematics by measuring proximal-distal (ulnocarpal impaction) and palmar-dorsal (DRUJ stability) translations using four 3-D parameters. This quantitative method may improve diagnostic consistency and reduce inter-observer variability inherent to manual measurements. Unlike conventional assessments focused only on PS (Shakoor et al., 2019; Wijffels et al., 2016), our approach evaluated wrist kinematics across RUD, FE and PS, establishing normal 3-D values for all motions. The integration of 3-D UV and UcP for the lunate and triquetrum enabled a comprehensive evaluation of ulnocarpal impaction, while the 3-D mru and 3-D epi offered a detailed 3-D assessment of DRUJ stability, overcoming the limitations of conventional 2-D methods.

In the present study, median 3-D UV in neutral wrist position was −0.4 mm (−1.5 to 0.3), which is similar to previous radiographic studies reporting UV values ranging from −0.3 mm to 0.7 mm in normal wrists (Freedman et al., 1998; Jung et al., 2001; Schuurman et al., 2001). In this study, dynamic assessment revealed that the 3-D UV remained relatively stable during RUD but varied during FE and PS, emphasising its dependence on wrist position. Notably, we did not observe a distinct increase in 3-D UV during pronation, contrasting with previous radiography findings (Epner et al., 1982; Tomaino, 1998). However, our results align with those of Kawanishi et al. (2014), who compared conventional 2-D radiographic measurements of UV with CT-based 3-D measurements and found that, contrary to the 2-D results, 3-D UV decreased in pronation. They attributed this to projection inaccuracies inherent to 2-D imaging, such as beam incident angle and positioning artefacts. These factors may also explain the decrease in 3-D UV during pronation observed in our study.

The UcP calculated the shortest distance from the ulna to the carpal bones. When interpretating the results, it is important to recognize that the location of this measurement may vary per wrist position, depending on where the shortest distance is measured (see Supplementary files 1–3). In neutral wrist position and during most movements, the median UcP-T was larger than the UcP-L. While the UcP-L remained relatively stable throughout motion, the UcP-T varied more, decreasing in pronation and ulnar deviation, findings consistent with previous fluoroscopic assessment by Logli et al. (2023) and the 3-D analysis by Shin et al. (2019). In contrast to the UcP-T, the median UcP-L was smaller in radial than in ulnar deviation, aligning with Logli et al. (2023) and Park et al. (2024). Our study observed no clear difference in median UcP-L between pronation and supination, consistent with Kawanishi et al. (2014) who did not find a statistically significant difference between pronation and supination. In contrast, Park et al. (2024) reported larger UcP-L values in pronation, possibly due to their weightbearing PS method.

In the present study, median 3-D mru shifted from 11.9% (−2.8 to 20.3) in supination to −11.2% (−20.8 to −0.2) in pronation, indicating a dorsal-to-palmar translation of the radius during this movement, consistent with previous 2-D CT studies of asymptomatic wrists (Park and Kim, 2008; Shakoor et al., 2019; Wijffels et al., 2016). During FE and RUD, median 3-D mru remained stable, indicating minimal palmar-dorsal translation at the DRUJ in normal wrists. The 3-D epi results showed a similar pattern, although the Δ3-D epi during PS was smaller. While previous studies found a dorsal position of the ulnar epicentre in supination (Park and Kim, 2008; Shakoor et al., 2019; Wijffels et al., 2016), we observed a palmar position of this point in supination. This discrepancy probably stems from differences in measurement methods, as our automated 3-D approach defined the ulnar epicentre differently than conventional methods.

Several limitations should be considered. First, despite adequate participant instruction and practice of movements prior to imaging, the range of wrist motion during imaging varied due to the freehand execution, limiting comparisons at extreme wrist positions as only few wrists achieved these positions. To illustrate this effect, the dashed vertical lines in Figures 7–10 indicate the range of motion that was achieved by at least 22 wrists. Furthermore, the effect of CT scanning parameters on workflow performance was not analysed. The use of data from two studies, one employing unilateral and the other bilateral dynamic scans with a relatively larger field of view, resulted in voxel size variability, which may have influenced the results. Third, although the automated method for anatomical landmark selection ensures consistency, the impact of landmark selection on kinematics was not analysed. Additionally, data from a single institution and single type of CT scanner were used in this study. To facilitate clinical implementation of dynamic CT imaging internationally, the proposed workflow should be validated using data from multiple institutions and scanner types. Finally, the relatively small sample size (*n* = 30 per wrist movement), limited subgroup analyses based on sex, age, or hand dominance. Future studies with larger cohorts should explore whether these factors influence ulnar-sided wrist kinematics.

This study provided normal values for ulnar-sided wrist kinematics in normal wrists. Future studies should include patients experiencing ulnar-sided wrist pain and comparing their outcomes with the normal data. Such comparisons may clarify whether the identified parameters can reliably distinguish pathological from normal wrists. The relatively large IQR observed suggest considerable variability in proximal-distal and palmar-dorsal ulnar translation during wrist motion in normal wrists. This variability probably reflects the complex interplay of anatomical, biomechanical, and functional differences, including ligament tension and soft tissue laxity (Omokawa et al., 2017). Detecting subtle abnormalities through dynamic CT imaging may help diagnosing ulnocarpal impaction and DRUJ instability. As dynamic CT imaging allows for bilateral analysis, intra-patient comparisons between affected and normal wrists may improve the diagnostic accuracy and reveal patient-specific wrist kinematics (Wijffels et al., 2016).

## Supplemental Material

sj-gif-1-jhs-10.1177_17531934251397297 – Supplemental material for Dynamic CT-based assessment of ulnar-sided wrist kinematics in healthy participants using automated 3-D analysis Supplemental material, sj-gif-1-jhs-10.1177_17531934251397297 for Dynamic CT-based assessment of ulnar-sided wrist kinematics in healthy participants using automated 3-D analysis by Erin Teule, Jesse Wolters, Nico Verdonschot, Stefan Hummelink and Brigitte van der Heijden in Journal of Hand Surgery (European Volume)

sj-gif-2-jhs-10.1177_17531934251397297 – Supplemental material for Dynamic CT-based assessment of ulnar-sided wrist kinematics in healthy participants using automated 3-D analysis Supplemental material, sj-gif-2-jhs-10.1177_17531934251397297 for Dynamic CT-based assessment of ulnar-sided wrist kinematics in healthy participants using automated 3-D analysis by Erin Teule, Jesse Wolters, Nico Verdonschot, Stefan Hummelink and Brigitte van der Heijden in Journal of Hand Surgery (European Volume)

sj-gif-3-jhs-10.1177_17531934251397297 – Supplemental material for Dynamic CT-based assessment of ulnar-sided wrist kinematics in healthy participants using automated 3-D analysis Supplemental material, sj-gif-3-jhs-10.1177_17531934251397297 for Dynamic CT-based assessment of ulnar-sided wrist kinematics in healthy participants using automated 3-D analysis by Erin Teule, Jesse Wolters, Nico Verdonschot, Stefan Hummelink and Brigitte van der Heijden in Journal of Hand Surgery (European Volume)

sj-gif-4-jhs-10.1177_17531934251397297 – Supplemental material for Dynamic CT-based assessment of ulnar-sided wrist kinematics in healthy participants using automated 3-D analysis Supplemental material, sj-gif-4-jhs-10.1177_17531934251397297 for Dynamic CT-based assessment of ulnar-sided wrist kinematics in healthy participants using automated 3-D analysis by Erin Teule, Jesse Wolters, Nico Verdonschot, Stefan Hummelink and Brigitte van der Heijden in Journal of Hand Surgery (European Volume)

sj-gif-5-jhs-10.1177_17531934251397297 – Supplemental material for Dynamic CT-based assessment of ulnar-sided wrist kinematics in healthy participants using automated 3-D analysis Supplemental material, sj-gif-5-jhs-10.1177_17531934251397297 for Dynamic CT-based assessment of ulnar-sided wrist kinematics in healthy participants using automated 3-D analysis by Erin Teule, Jesse Wolters, Nico Verdonschot, Stefan Hummelink and Brigitte van der Heijden in Journal of Hand Surgery (European Volume)

sj-gif-6-jhs-10.1177_17531934251397297 – Supplemental material for Dynamic CT-based assessment of ulnar-sided wrist kinematics in healthy participants using automated 3-D analysis Supplemental material, sj-gif-6-jhs-10.1177_17531934251397297 for Dynamic CT-based assessment of ulnar-sided wrist kinematics in healthy participants using automated 3-D analysis by Erin Teule, Jesse Wolters, Nico Verdonschot, Stefan Hummelink and Brigitte van der Heijden in Journal of Hand Surgery (European Volume)
