# Supplementary figures and images for: Dynamic CT-based assessment of ulnar-sided wrist kinematics in healthy participants using automated 3-D analysis
Source: J Hand Surg Eur Vol. 2025 Nov 21;51(4):488–98. doi: 10.1177/17531934251397297 (PMC12967434; doi:10.1177/17531934251397297)

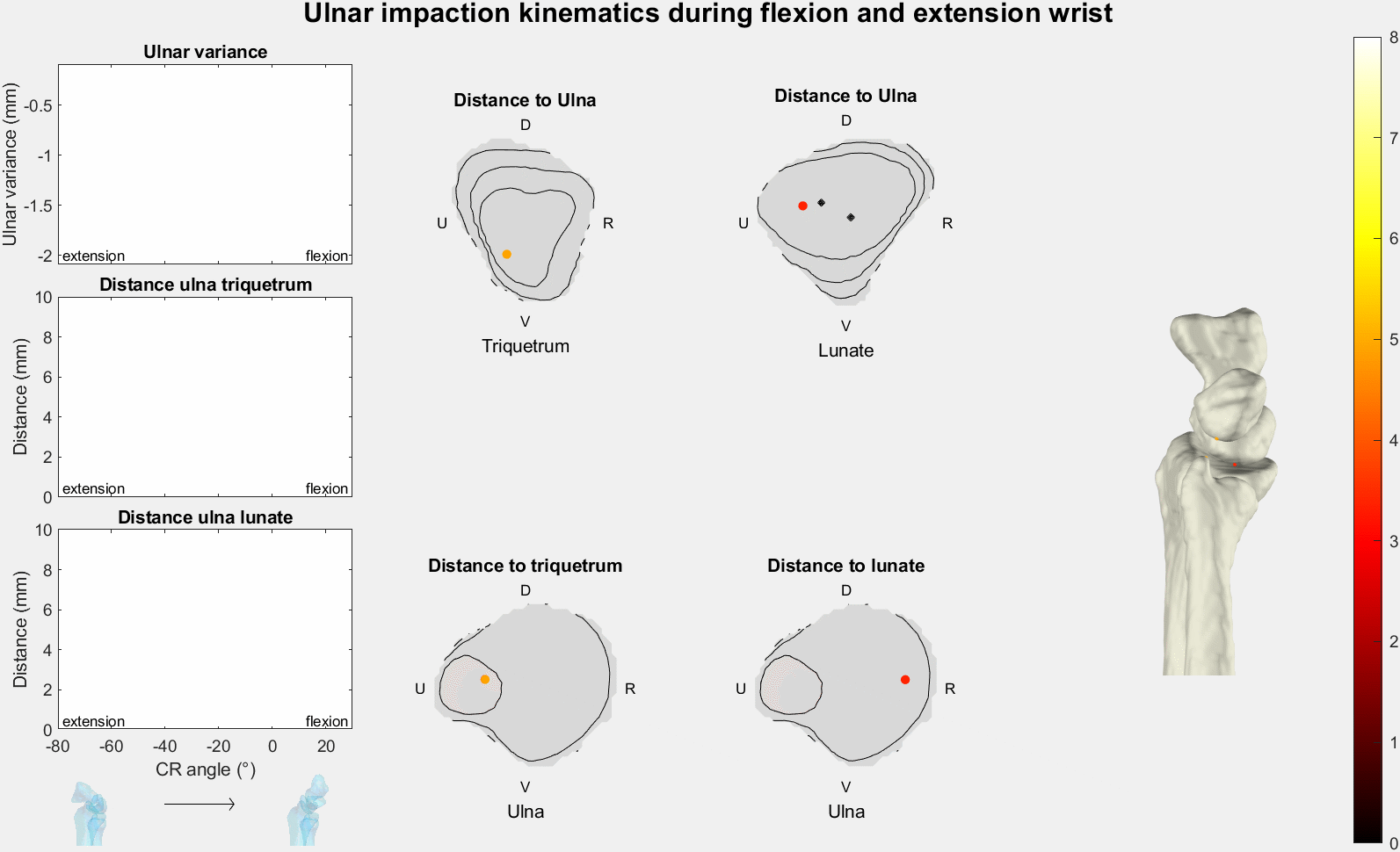

Supplement: sj-gif-1-jhs-10.1177_17531934251397297 – Supplemental material for Dynamic CT-based assessment of ulnar-sided wrist kinematics in healthy participants using automated 3-D analysis [file sj-gif-1-jhs-10.1177_17531934251397297.gif]

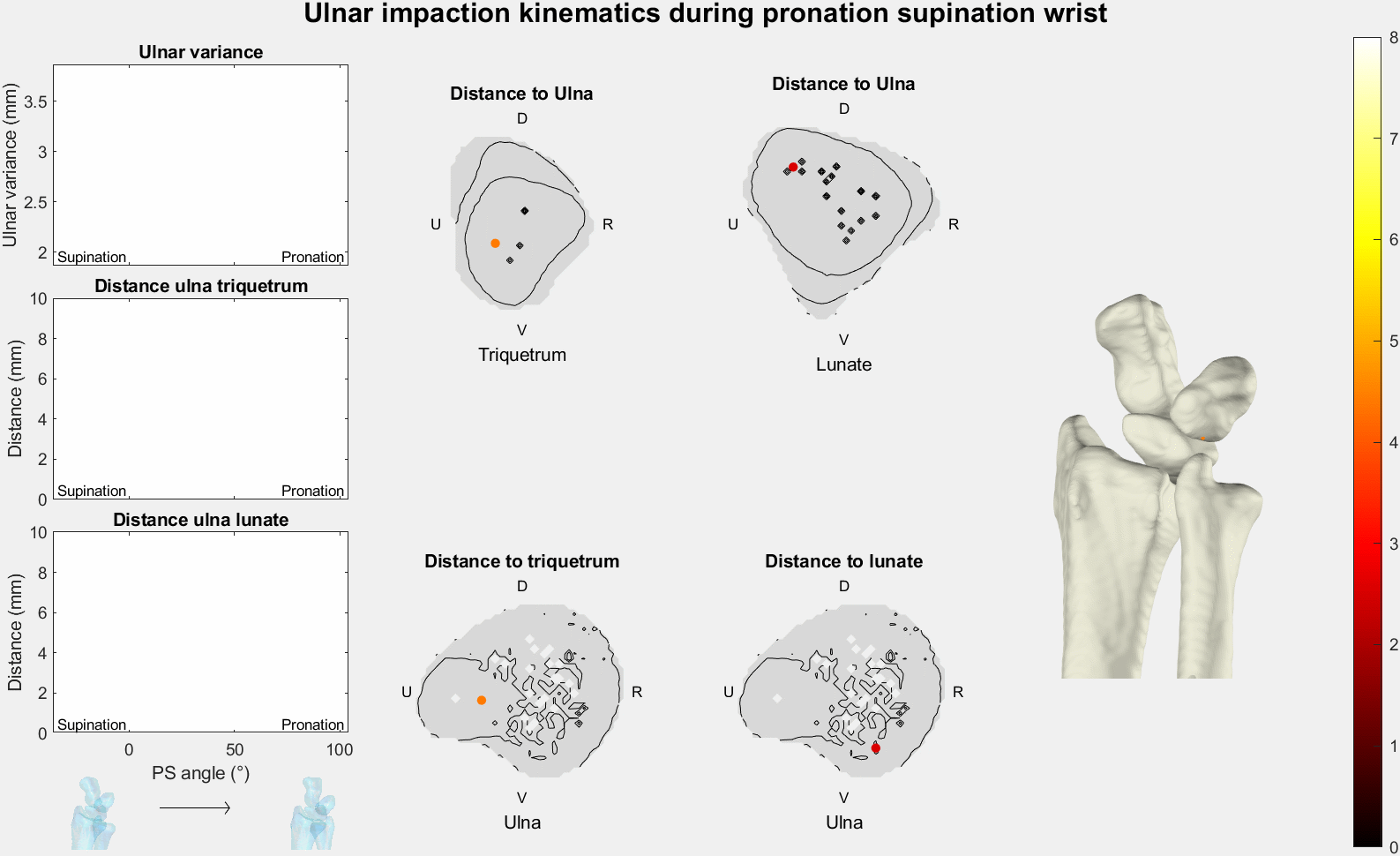

Supplement: sj-gif-2-jhs-10.1177_17531934251397297 – Supplemental material for Dynamic CT-based assessment of ulnar-sided wrist kinematics in healthy participants using automated 3-D analysis [file sj-gif-2-jhs-10.1177_17531934251397297.gif]

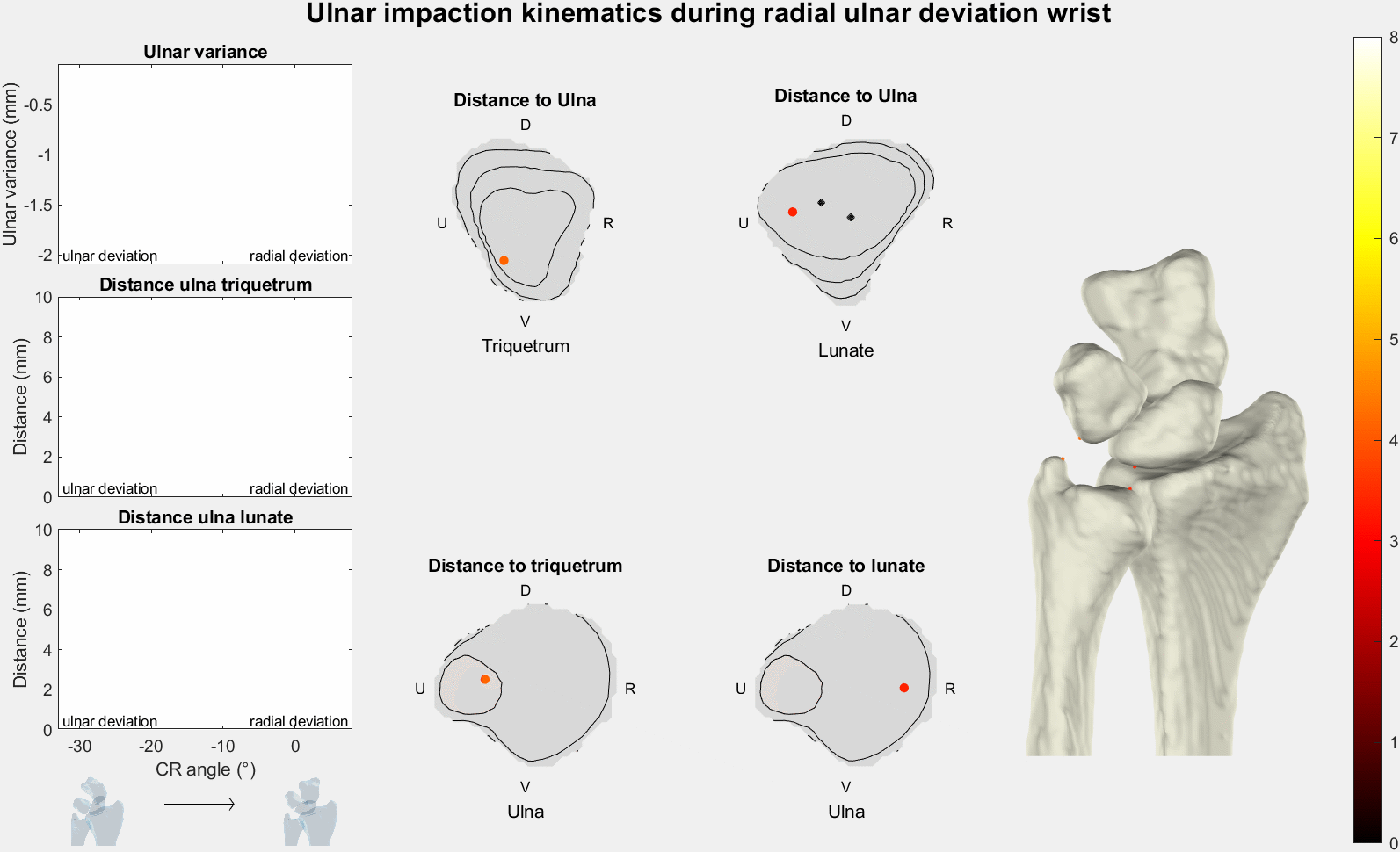

Supplement: sj-gif-3-jhs-10.1177_17531934251397297 – Supplemental material for Dynamic CT-based assessment of ulnar-sided wrist kinematics in healthy participants using automated 3-D analysis [file sj-gif-3-jhs-10.1177_17531934251397297.gif]

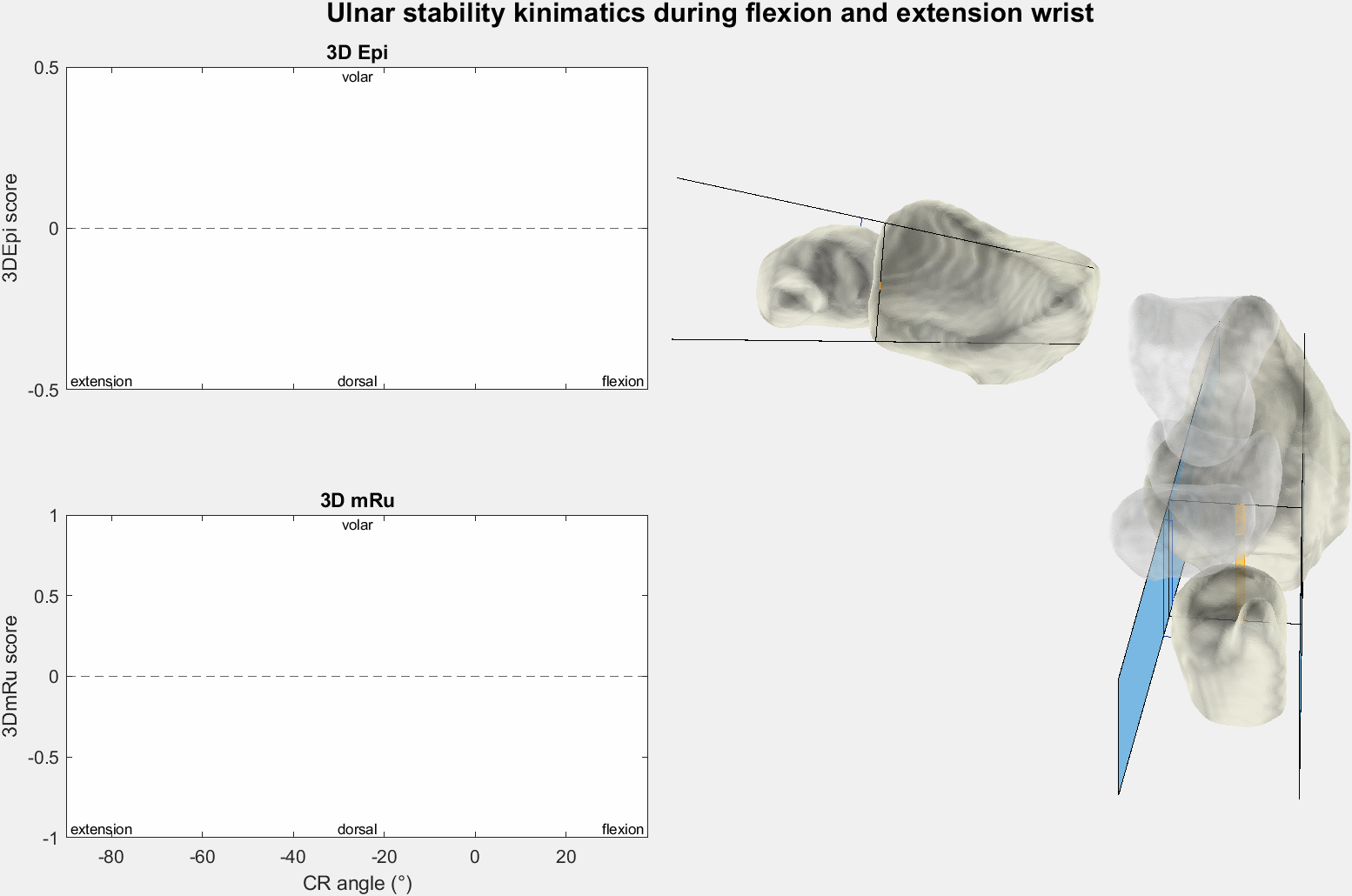

Supplement: sj-gif-4-jhs-10.1177_17531934251397297 – Supplemental material for Dynamic CT-based assessment of ulnar-sided wrist kinematics in healthy participants using automated 3-D analysis [file sj-gif-4-jhs-10.1177_17531934251397297.gif]

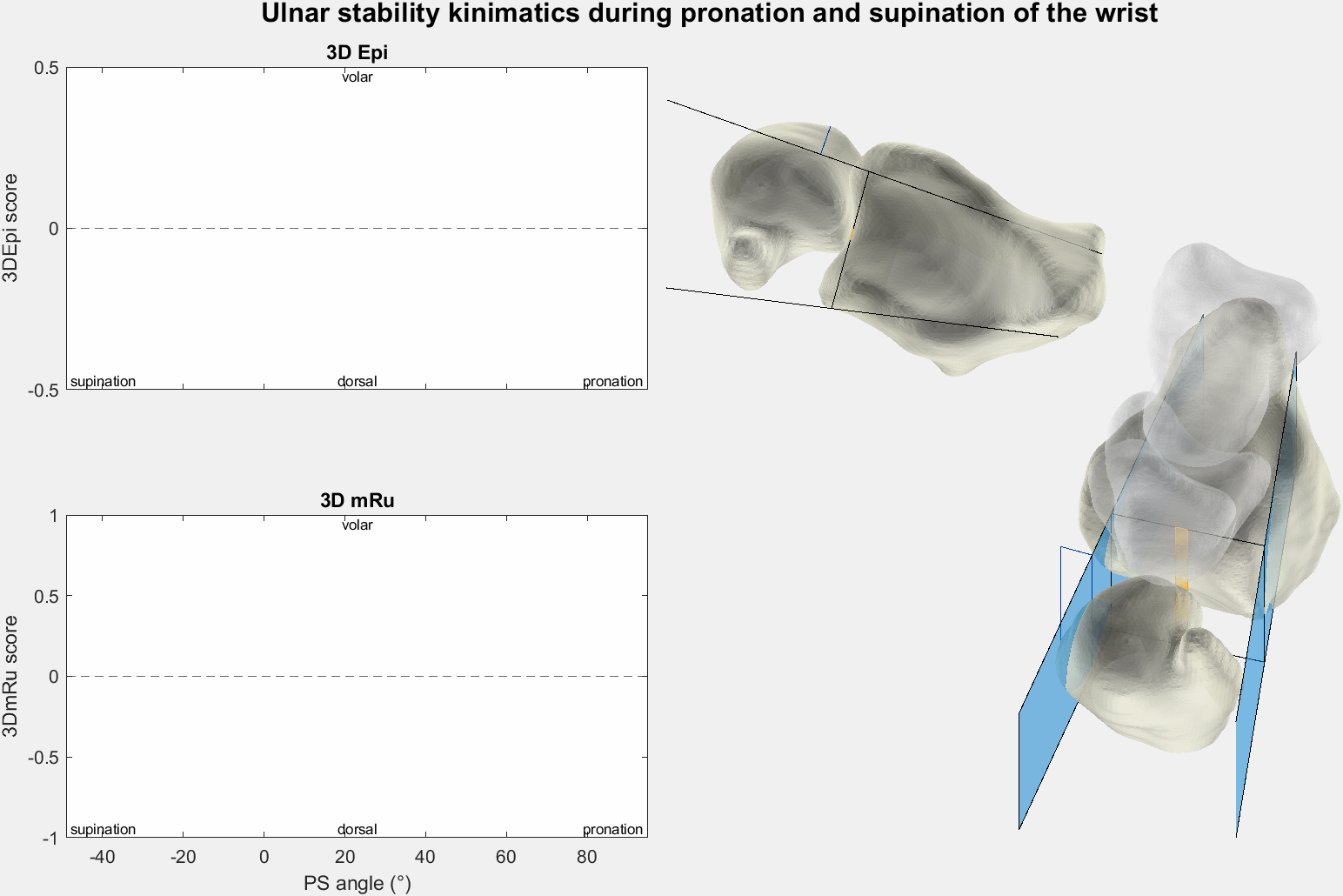

Supplement: sj-gif-5-jhs-10.1177_17531934251397297 – Supplemental material for Dynamic CT-based assessment of ulnar-sided wrist kinematics in healthy participants using automated 3-D analysis [file sj-gif-5-jhs-10.1177_17531934251397297.gif]

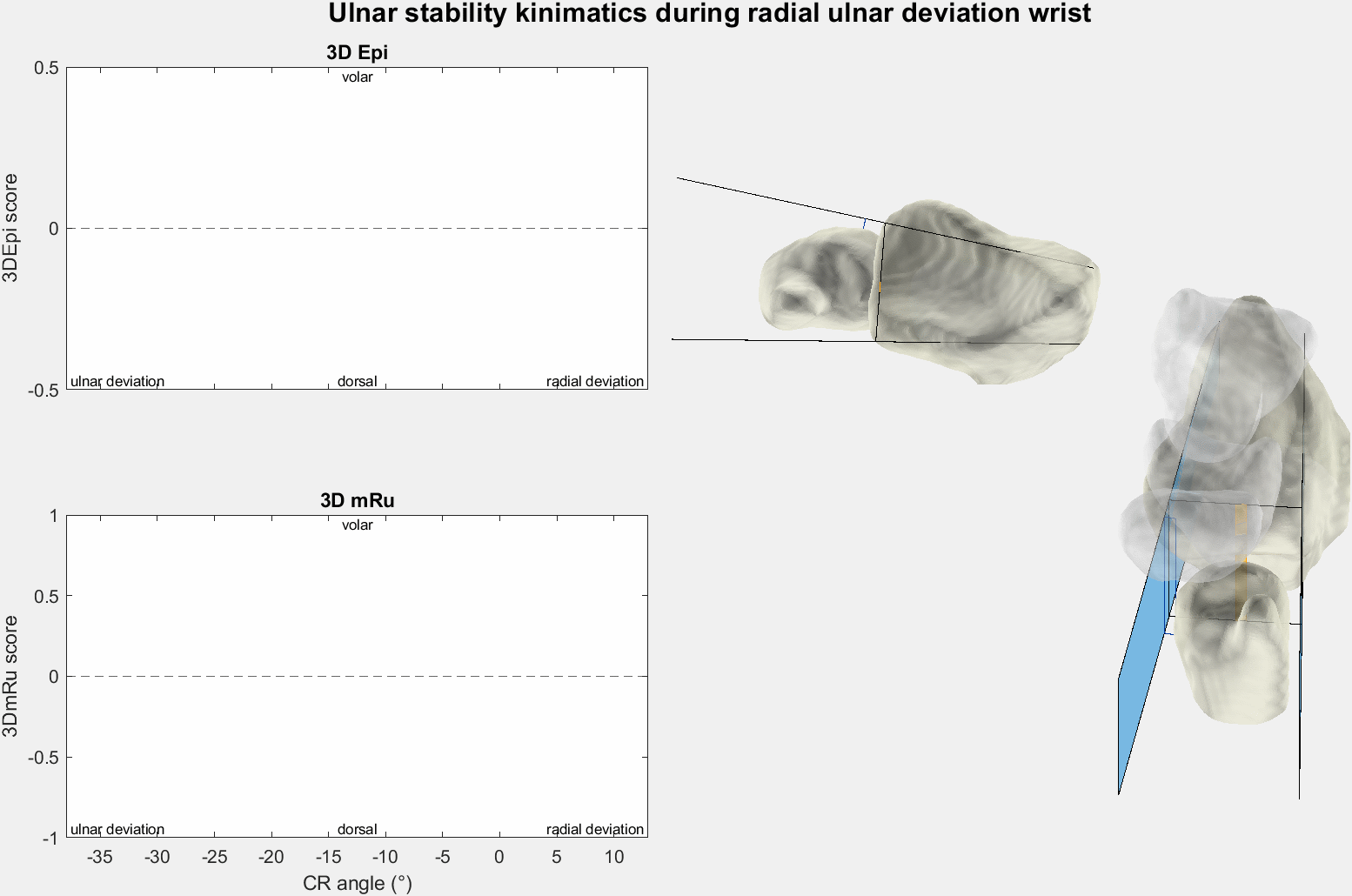

Supplement: sj-gif-6-jhs-10.1177_17531934251397297 – Supplemental material for Dynamic CT-based assessment of ulnar-sided wrist kinematics in healthy participants using automated 3-D analysis [file sj-gif-6-jhs-10.1177_17531934251397297.gif]
